# 117. How Does Antimicrobial Stewardship Provider Role Affect Prospective Audit and Feedback Acceptance by the Attending Physician?

**DOI:** 10.1093/ofid/ofab466.319

**Published:** 2021-12-04

**Authors:** Keely Hammond, Justin Chen, Karen Doucette, Stephanie Smith, Dima Kabbani, Cecilia Lau, Serena Bains, Jackson J Stewart, Karen G Fong

**Affiliations:** 1 University of Alberta, Edmonton, Alberta, Canada; 2 Alberta Health Services, Edmonton, Alberta, Canada; 3 University of Alberta Hospital, Edmonton, Alberta, Canada

## Abstract

**Background:**

Antimicrobial stewardship (AMS) teams are commonly multidisciplinary. The effect of AMS provider role on prospective audit and feedback (PAF) acceptance has previously been investigated with mixed results. PAF of restricted antimicrobials (carbapenems, linezolid, daptomycin, and tigecycline) in adult inpatients at our large Canadian academic centre has been performed since 2018. Actionable feedback is communicated via chart note plus one of a phone call, direct message, or in-person discussion with the most responsible physician of the attending team in order to optimize the prescription if deemed necessary. The objective of this study was to assess the effect of AMS provider role on PAF acceptance.

**Methods:**

A 3 year retrospective review of all PAF events was undertaken. All audited prescriptions were included. Logistic regression was used to determine odds ratios for acceptance for individual AMS provider roles of pharmacist, physician, and supervised post-graduate physician trainee.

**Results:**

Out of 1896 prescriptions audited, actionable feedback was provided to the most responsible physician in 731 (39%) cases. 677/731 (93%) of audited antibiotics were carbapenems. The overall acceptance rate was 82% (598/731). Acceptance rate and odds of acceptance based on AMS provider role were as follows: pharmacist alone 171/208 (82%), OR 1.04, 95% CI 0.70-1.59, physician alone 141/160 (88%), OR 1.85, 95% CI 1.12-3.20, pharmacist-physician duo 211/268 (79%), OR 0.73, 95% CI 0.50-1.07, and supervised post-graduate physician trainee 75/95 (79%), OR 0.81, 95% CI 0.48-1.41.

**Conclusion:**

The overall acceptance rate was high. There was a higher odds of acceptance if an AMS physician was providing PAF alone, highlighting the importance of physician involvement.

**Disclosures:**

**Dima Kabbani, MD**, **AVIR Pharma** (Grant/Research Support, Other Financial or Material Support, Speaker)**Edesa Biotech** (Scientific Research Study Investigator)**Merck** (Scientific Research Study Investigator)

